# Targeting surface nucleolin with a multivalent pseudopeptide delays development of spontaneous melanoma in RET transgenic mice

**DOI:** 10.1186/1471-2407-10-325

**Published:** 2010-06-24

**Authors:** Diala El Khoury, Damien Destouches, Renée Lengagne, Bernard Krust, Yamina Hamma-Kourbali, Marylène Garcette, Sandra Niro, Masashi Kato, Jean-Paul Briand, José Courty, Ara G Hovanessian, Armelle Prévost-Blondel

**Affiliations:** 1 UPR 2228 CNRS, Université Paris Descartes, 45 rue des Saints Pères, 75270 Paris Cedex 06, France; 2 EAC 7149 CNRS, Université Paris-Est, 61 avenue du général de Gaulle, 94000 Créteil, France; 3 Institut Cochin, Université Paris Descartes, CNRS (UMR 8104), 27 rue du Faubourg Saint-Jacques, 75014 Paris, France; 4 INSERM U1016, Paris, France; 5 Unit of Environmental Health Sciences, Department of Biomedical Sciences, College of Life and Health Sciences, Chubu University, 1200 Matsumoto-cho, Kasugai-shi, Aichi 487-8501, Japan; 6 UPR 9021 CNRS, IBMC, 15 rue René Descartes, Strasbourg, France; 7 Armelle Prévost-Blondel, Institut Cochin, Département Immunologie/Hématologie, 27 rue du Faubourg Saint-Jacques, Paris, F-75014 France

## Abstract

**Background:**

The importance of cell-surface nucleolin in cancer biology was recently highlighted by studies showing that ligands of nucleolin play critical role in tumorigenesis and angiogenesis. By using a specific antagonist that binds the C-terminal tail of nucleolin, the HB-19 pseudopeptide, we recently reported that HB-19 treatment markedly suppressed the progression of established human breast tumor cell xenografts in the athymic nude mice without apparent toxicity.

**Methods:**

The *in vivo *antitumoral action of HB-19 treatment was assessed on the spontaneous development of melanoma in the RET transgenic mouse model. Ten days old RET mice were treated with HB-19 in a prophylactic setting that extended 300 days. In parallel, the molecular basis for the action of HB-19 was investigated on a melanoma cell line (called TIII) derived from a cutaneous nodule of a RET mouse.

**Results:**

HB-19 treatment of RET mice caused a significant delay in the onset of cutaneous tumors, several-months delay in the incidence of large tumors, a lower frequency of cutaneous nodules, and a reduction of visceral metastatic nodules while displaying no toxicity to normal tissue. Moreover, microvessel density was significantly reduced in tumors recovered from HB-19 treated mice compared to corresponding controls. Studies on the melanoma-derived tumor cells demonstrated that HB-19 treatment of TIII cells could restore contact inhibition, impair anchorage-independent growth, and reduce their tumorigenic potential in mice. Moreover, HB-19 treatment caused selective down regulation of transcripts coding matrix metalloproteinase 2 and 9, and tumor necrosis factor-α in the TIII cells and in melanoma tumors of RET mice.

**Conclusions:**

Although HB-19 treatment failed to prevent the development of spontaneous melanoma in the RET mice, it delayed for several months the onset and frequency of cutaneous tumors, and exerted a significant inhibitory effect on visceral metastasis. Consequently, HB-19 could provide a novel therapeutic agent by itself or as an adjuvant therapy in association with current therapeutic interventions on a virulent cancer like melanoma.

## Background

Nucleolin is an abundant DNA-, RNA- and protein-binding protein ubiquitously expressed in exponentially growing eukaryotic cells. It is found at several locations in cells: in the nucleolus it controls many aspects of DNA and RNA metabolism; in the cytoplasm it shuttles proteins into the nucleus and provides a posttranscriptional regulation of strategic mRNAs; and on the cell surface it serves as an attachment protein for several ligands from growth factors to microorganisms [1-7]. Surface and cytoplasmic nucleolin are differentiated from nuclear nucleolin by a slight shift in their isoelectric point, which could reflect glycosylation of surface/cytoplasmic nucleolin [3,8,9]. Moreover, surface/cytoplasmic nucleolin is regulated independently of its nuclear counterpart, since marked reduction of surface/cytoplasmic nucleolin could occur without any apparent effect on the level or nucleolar localization of nuclear nucleolin [10].

Emerging evidences highlight the importance of the cell-surface expressed nucleolin in cell proliferation, tumor cell growth and angiogenesis [3,10-14]. The enhanced expression of surface nucleolin is observed *in vitro *and *in vivo *in lymphoid organs containing activated lymphocytes, on the surface of tumor cells and activated endothelial cells, or in angiogenic endothelial cells within the tumor vasculature [11,14,15]. Among surface nucleolin binding growth factors and proteins, midkine and pleiotrophin can transform cells, whereas on endothelial cells they exert both mitogenic and angiogenic effect [16]. Urokinase that is implicated in mechanisms regulating pericellular proteolysis, cell-surface adhesion, and mitogenesis binds and is co-internalized with surface nucleolin [17,18]. Other surface nucleolin binding proteins such as laminin-1, factor J, L- and P-selectins, and hepatocyte growth factor are involved in tumor development, induce cell differentiation, regulate cell adhesion, leukocyte trafficking, inflammation and angiogenesis [19-23]. The tumor homing peptide F3 that binds both endothelial and tumor cells is internalized via surface nucleolin, while endostatin that inhibits angiogenesis binds nucleolin on the surface of endothelial cells before translocation to the nucleus [11,13]. Accordingly, the functional blockade or down-regulation of surface nucleolin in endothelial cells inhibits migration of endothelial cells and prevents capillary-tubule formation [10,12]. Ligand binding results in clustering of cell-surface nucleolin in lipid raft membrane microdomains before endocytosis of the ligand-nucleolin complex by an active process [5,24,25].

We recently reported that both tumor growth and angiogenesis could be suppressed by targeting surface nucleolin using the HB-19 pseudopeptide, which binds the RGG domain located at the C-terminal tail of nucleolin [10,26]. HB-19 reduced markedly colony-forming capacity of several human carcinoma cell lines in soft agar, impaired migration of endothelial cells and formation of capillary-like structures in collagen gel, and reduced vessel arborization in the chick embryo chorioallantoic membrane. Significantly, HB-19 treatment markedly suppressed the progression of established human breast tumor cell xenografts in athymic nude mice, and in some cases eliminated measurable tumors while displaying no toxicity to normal tissue [10].

In a more relevant tumor model, now we provide evidence that HB-19 can also interfere with the spontaneous development of cancer in RET mice. Such mice express constitutively an active form of the *ret *oncogene leading to development of spontaneous melanoma, thus providing a genetically driven model of tumors [27]. In this model the severity grade of melanoma is associated with the location of skin tumors in which the onset of dorsal nodules corresponds to a more aggressive disease [28]. The skin primary tumors eventually metastasize mainly to lymph nodes, mediastinum or lungs [27]. Moreover, our recent data in this model suggests that the growth of melanoma involved several tolerance mechanisms [29]. Here we show that HB-19 treatment delays significantly the onset and frequency of spontaneous melanoma in RET mice. In addition, the frequency of visceral metastasis and tumor vascularization are significantly reduced in HB-19 treated compared to control mice, thus indicating inhibitory effects on both metastasis and angiogenesis. Using a melanoma cell line derived from a cutaneous nodule of a RET mouse, we show that HB-19 can affect several criteria implicated in the tumorigenic potential of melanoma cells, such as restoration of contact inhibition in culture, reduction of colony formation in soft agar, and impairment of tumorigenicity and lung metastasis in mice. Interestingly, these changes are associated with a specific inhibitory action of HB-19 on expression of genes implicated in tumorigenesis.

## Methods

### Mice

MT/*ret*^+/- ^transgenic mice (C57BL/6 background, called RET mice, litter one) expressing the *rfp-ret *oncogene develop a spontaneous melanoma [27,30]. Constitutively activated *rfp-ret *enhances cRET protein expression in the process of melanomagenesis in RET mice [31]. Non-transgenic littermates (MT/*ret*^-/-^) were used for transplantation experiments. Mice were maintained in our own animal facilities corresponding to a pathogen free environment. All experiments were performed in compliance with French Ministry of Agriculture regulations for animal experimentation (number 75-510).

### HB-19 treatment of RET mice

HB-19 was synthesized as described previously [10,26]. Although it is readily soluble in water, HB-19 was dissolved in PBS for the purpose of treatment of mice. Ten days old RET mice were treated intraperitoneally with HB-19 at 5 injections/week during week 1-3 and 2 injections/week during week 4-42. The dose of HB-19 was 50, 100, and 200 μg for the first, second and the rest of the weeks, respectively. Control mice were injected with PBS at the different time points. Clinical signs of mice treated (n = 9) and untreated (n = 11) were assessed once a week on vigil mice and once a month on anesthetized mice. Development of facial or dorsal tumor nodules was recorded. At the end of the treatment (day 300), all mice were sacrificed and autopsied to monitor for distant metastasis.

**Flow cytometry**. Cutaneous tumors were pooled, mechanically dissociated and digested with 1 mg/mL collagenase A and 0.1 mg/mL DNase I (Roche, Mannheim, Germany) for 25 min at 37°C. Single cell suspensions were filtered, washed in PBS, 5% FCS, 0.5 mM EDTA and resuspended in RPMI 1640. After incubation with anti-FcgII/IIIR antibody (clone 2.4G2), cell suspensions were stained at 4°C, for 15 min with the following combinations of monoclonal antibodies (all from Pharmingen): PerCP-conjugated anti-CD45.2/APC-conjugated anti-CD11b and APC-conjugated anti-CD45.2/PE-conjugated anti-TcRab. Flow cytometric analyses were performed on a FacsCalibur cytofluorometer (BD Biosciences) and data were analyzed using CellQuestPro Software (BD).

### Murine melanoma cells and transplantation

The TIII cell-line was derived from the neck cutaneous nodule that developed in a RET mouse. Cells were cultured in RPMI 1640 with 10% FCS, 2 mM L-glutamine, 100 U/ml penicillin, 100 μg/ml streptomycin. In addition to the nucleus, nucleolin is also expressed in the cytoplasm and at the cell surface of TIII cells, as it is the case in different types of tumor cells and other melanoma cell lines [3,32].

TIII (1×10^6^) cells cultured in the presence or not of HB-19 (10 μM; 12 passages) were transplanted subcutaneously in 10 weeks old MT/*ret*^-/- ^mice. Fourteen days later, mice were sacrificed and tumor mass was determined with a caliper. Alternatively, mice were injected in the tail vein with TIII cells (5×10^5^) in the absence or presence of 10 μM of HB-19. Twenty hours later and then daily during two weeks, mice were treated by intraperitoneal injections of PBS or PBS containing HB-19 (5 mg/kg). Mice were then sacrificed, and the number of black macro-metastases on the lung surface was counted.

### Immunofluorescence and confocal microscopy

Cells were plated 24 hours before the experiment in eight-well glass slides (Lab-Tek Brand; Nalge Nunc International, Naperville, IL). Cells were fixed with paraformaldehyde (PFA; 3.7%, 10 min), permeabilized by Triton X-100 (0.5%, 15 min) and stained for the intracellular actin cytoskeleton using FITC-conjugated phalloidin (Sigma) [10,26]. The nuclei were stained with 4',6-diamidino-2-phenylindole (DAPI).

### Colony formation in soft agar

TIII cells (2×10^4^) were mixed in 0.35% top agar diluted in RPMI containing 10% FCS in the absence or presence of different concentrations of HB-19 before plating onto 0.8% bottom agar in 12-multiwell plates. Cells were treated every two days during 14 days. Colonies with diameter superior to 100 μm were scored as positive using a phase contrast microscope equipped with a measuring grid at magnification 50×. The number of colonies was determined by analyzing 5 fields/well from 3 wells [10].

### Tissue preparation, immunohistochemical staining and image analysis

Tumors were fixed in FineFix (Milestone, Bazainville, France) for paraffin inclusion. Sections of 8 μm thickness were re-hydrated and saturated in PBS containing 5% goat serum. Sections were incubated with 1:20 dilution of a rat anti-mouse CD34 monoclonal antibody (Abcam, Cambridge, UK) for 2 hours at room temperature. After two washes in PBS, sections were incubated for 1 hour at room temperature with biotin-conjugated goat anti-rat IgG (Chemicon International Inc., Temecula, CA) diluted at 1:500, followed by three washes in PBS and incubation with avidin/peroxidase complex (Vector Laboratories, Burlingame, CA). The horseradish-Peroxidase activity was revealed by incubating the sections with 3,3'-Diaminobenzidine substrate kit (Vector Laboratories). Finally, the sections were counterstained with haematoxylin, followed by water wash and cover slipped with Mowiol medium. Five microscopic fields (at 200-fold magnification) were selected randomly for analysis using the Image analysis. The density of endothelial cells in each field was expressed as the ratio of cell area/total area examined × 100 (%). These values were then averaged for the tumors recovered from control and HB-19 treated mouse.

### mRNA expression monitored by RT-PCR

TIII cells were cultured in RPMI medium containing 10% FCS in the absence or presence of HB-19. Total RNA was prepared from cells (5×10^5^) and fresh tumors isolated from control and HB-19 treated RET mice using RNeasy Mini Kit (Qiagen) according to the manufacturer's instructions. RT was carried out with oligo(dT) and 1-4 μg of total RNA using Superscript II RNase H- Reverse Transcriptase (Gibco BRL). The expression of specific mRNAs was investigated by RT-PCR using primers for matrix metalloproteinase-2 and -9 (MMP-2, MMP-9), vascular endothelial growth factor (VEGF-A), tumor necrosis factor alpha (TNF-α), signal transducer and activator of transcription 1 (STAT-1), melanoma inhibitory activity (MIA), and glyceraldehyde-3-phosphate dehydrogenase (GAPDH). PCR was performed in a RoboCycler 96 (Stratagene) with the following primers: MMP-2 sense 5'-GAGTTGGCAGTGCAATACCT-3' and antisense 5'-GCCGTCCTTCTCAAAGTTGT-3'; MMP-9 sense 5'-AGTTTGGTGTCGCGGAGCAC-3' and antisense 5'-TACATGAGCGCTTCCGGCAC-3'; VEGF-A sense 5'-AGAGCAACATCACCATGCAG-3' and antisense 5'-AGGAATCCCAGAAACAACCC-3'; TNF-α sense 5'-ACTCCCAGAAAAGCAAGCAA-3' and antisense 5'-TGGAAGACTCCTCCCAGGTA-3'; STAT-1 sense 5'-CGTGGGAACGGAAGCATTTG-3 and antisense 5'-GAGACATCATAGGCAGCGTG-3'; MIA sense 5'-ATCCTATCTCCATGGCTGT-3' and antisense 5'-ACTGGCAGTAGAAATCCCA-3'; GAPDH sense 5'-CGTCCCGTAGACAAAATGGT-3' and antisense 5'-CCTTCCACAATGCCAAAGTT-3'. PCR amplification conditions were 95°C for 2 min, 30 cycles at 95°C for 30 sec, 53°C (for MMP-2, VEGF, Mia-1 and GAPDH) or 55°C (for STAT-1) or 57°C (MMP-9) for 30 sec and 72°C for 45 sec, and this was followed by 5-min incubation at 72°C. The expected RT-PCR product for MMP-2, MMP-9, VEGF-A, TNF-α, STAT-1, MIA-1 and GAPDH was 666, 754, 663, 688, 425, 267 and 527 base pairs, respectively.

### Statistical analysis

Statistical significance was determined by ANOVA unpaired T test or the Wilcoxon log-rank test using the GraphPad Prism 4.0 software (San Diego, CA). Values of p < 0.05 were considered significant.

## Results

### HB-19 delays significantly development of spontaneous melanoma in RET mice

**Figure 1:**
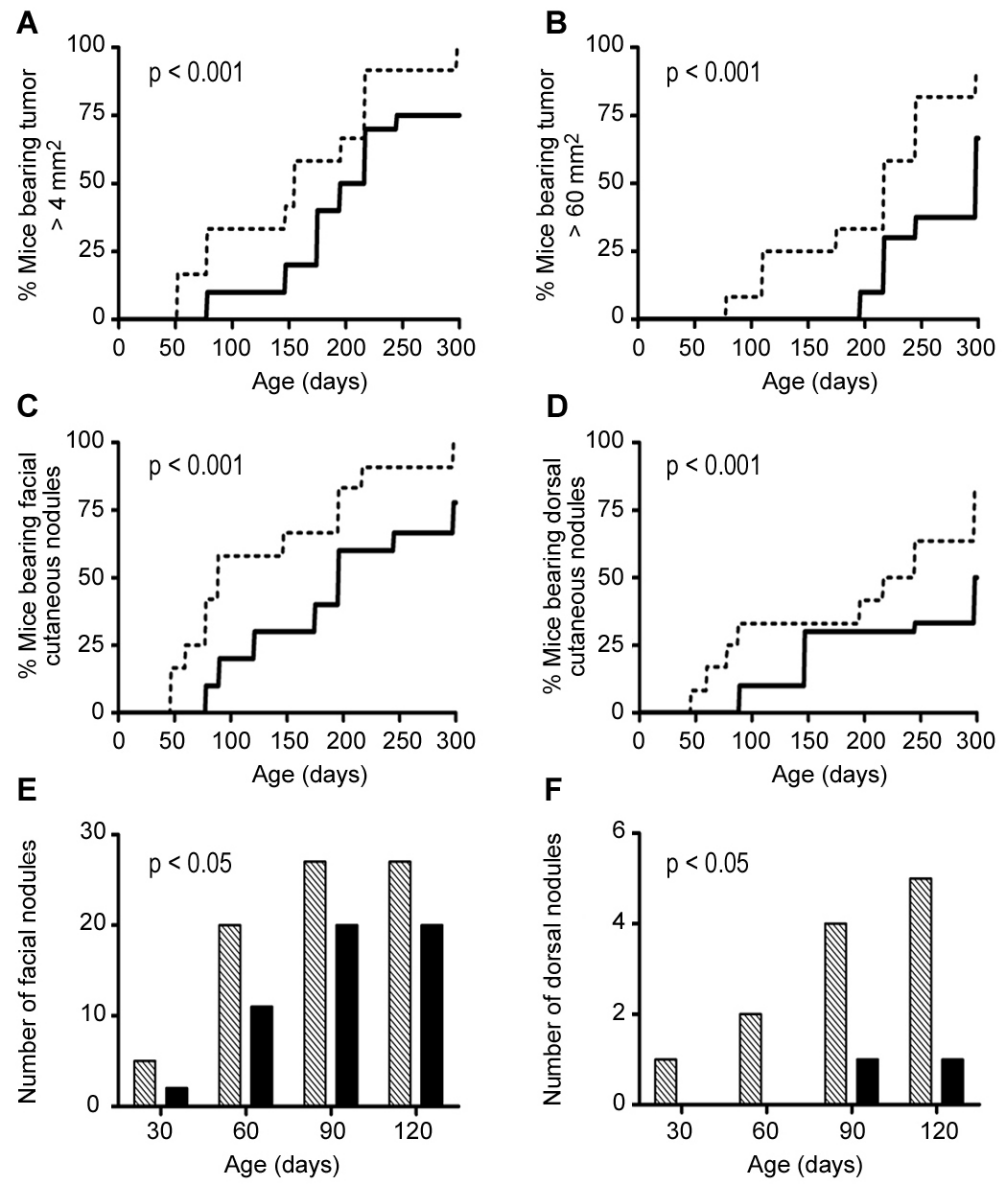
**HB-19 inhibits significantly spontaneous melanoma development**. RET mice were treated with HB-19 according to the schedule indicated in Methods and diagnosed frequently over a period of 300 days. A/B. HB-19 treatment delays significantly the onset of small cutaneous tumors (≥ 4 mm^2^) in panel A and of huge cutaneous tumors (> 60 mm^2^) in panel B (Log-rank Wilcoxon test, p < 0.001 for both graphs). The dotted lines correspond to data from untreated mice (n = 11) and full lines correspond to HB-19 treated mice (n = 9). C/D. HB-19 treatment significantly delays the incidence of facial (C) and dorsal (D) cutaneous nodules in RET mice. The dotted lines correspond to data from untreated mice and full lines correspond to HB-19 treated mice (Log-rank Wilcoxon test, p < 0.001). E/F. Number of facial (E) and dorsal (F) cutaneous nodules during the period of 120 days. The histograms show the progression of the number of nodules (calculated for 10 mice) in untreated (hatched bars) and HB-19 treated (black bars) mice, respectively. HB-19 treated mice displayed less cutaneous nodules than untreated mice (p < 0.05 for both graphs).

**Figure 2:**
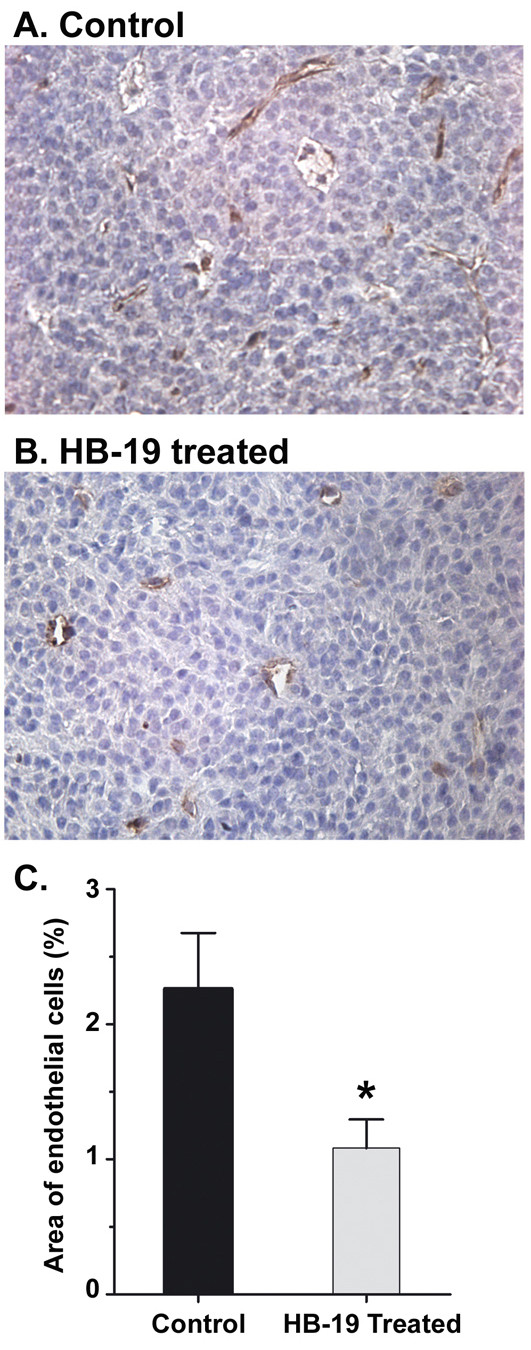
**Reduced density of blood vessels in tumors recovered from HB-19 treated RET mice**. Sections of tumors from control untreated (panel A; localized under the ear) and HB-19 treated (panel B; localized under the eye) RET mice were stained with antibodies against the CD34 endothelial marker and analyzed by fluorescence microscopy (Methods). Representative macroscopic images (magnification 200×) from each group of sections are presented. C. Quantification of angiogenesis was estimated by image analysis of CD34-labeled endothelial cells in tumor sections from control and HB-19 treated RET mice. Statistical significance: * 0.01 < p < 0.1.

To evaluate the *in vivo *anti-tumor effect of HB-19 on the natural course of melanoma progression, RET mice were treated in a prophylactic setting that extended 300 days as described in Methods. The particular aspects of the clinical diagnosis of control and HB-19 treated RET mice are presented individually in Table 1. HB-19 treatment significantly delayed the development of measurable cutaneous tumors that occurred at day 50 and 75 in control and treated mice, respectively (Figure 1A and 1B). The difference between these two groups was even more significant when the incidence of large cutaneous tumors (over 60 mm^2^) was compared (Log-rank test; p < 0.001). Indeed, large tumors were observed from day 75 onward in control mice, whereas they started to develop at day 190 in HB-19 treated mice (Figure 1B). We next examined the effect of HB-19 on all cutaneous nodules taking account their location, since our previous observations indicated that the severity grade of melanoma is associated with the location of skin tumors, and that in particular the occurrence of nodules in the posterior part of the body corresponds to a more aggressive disease [28]. Both facial and dorsal cutaneous nodules developed later and were less frequent in HB-19 treated compared to control mice (Figure 1C and 1D: Log-rank Wilcoxon test, p < 0.001; Figure 1E and 1F p < 0.05). In addition, tumor vascularization was evaluated by CD34 antigen staining in sections of tumors recovered from control and HB-19 treated RET mice (Figure 2). Clearly, numerous vessels remained with an open lumen in the control samples 'angiogenic hot spots' localized heterogeneously within the tumor as compared to HB-19 treated samples. Angiogenesis quantified by image analysis of CD34-labeled endothelial cells showed that HB-19 induced a reduction of 51% in microvessel density compared to control tumors.

**Table 1:** Particular aspects of the clinical diagnosis for each control and HB-19 treated RET mice

Treatment		Remarks in mice before autopsy (< Day 300)		Diagnosis at autopsy (Day 300)	
	
	Mice	Regression	Death	Cutaneous tumor location and size (mm)	Distant metastasis
Control	1			under eye 4, nose 3.1, under ear 4.9, back 6.7, 2.8 and 2.9, thigh 5.9	Mediastinal
	
	2			cheek 4.9, eye 6.2, under ear 5.4, 6.2, 7 and 5.7, back 6.3 and 4.2, around genitals 3.8 and 4.7	None
	
	3			cheek 3.3, under ear 6.7, around eye 6.3, back ear 3.5, back 3.2, thigh 5.1	Retroperitoneal
	
	4			under eye 6.7 and 2.3, cheek 4.1 and 3, upper ear 2.8, under ear 3.4 and 5.6, leg 4.9 and 4.3, genitals 4	Retroperitoneal
	
	5			under eye 11, 4 leg neighboring nodules 13.9	Visceral
	
	6			upper eye 6.3, under eye 2.6, between ears 5.4	Retroperitoneal
	
	7			cheek 2.9, nose 4.3, under ear 3.8, ear 4.7	Retroperitoneal
	
	8			cheek 3.1, under eye 2.7	None
	
	9			under ear 2.5, between eyes 3.8, under eye 6.3 and 3, leg 6.8, genitals 5.5, tail base 5.9	Retroperitoneal
	
	10			cheek 5.6, 5.1, 3.4 and 3.7, under eye 7.4, leg 4.7, thigh 11.3	None
	
	11			back ears 4.9, back 2.8, 1.9, 6.4, 3.5 and 2.2	Retroperitonel

HB-19 treated	1			under eye 6, cheek 3.5, around left eye 7.7, between ears 5, genitals 8, foot 9.2, tail 3.6	Visceral
	
	2	under eye and thigh		cheek 5.2 and 3, under eye 5, 3 nodules on the left cheek < 1, between ears 8.2, thigh 8.2, foot 7.9	Visceral
	
	3			cheek 2.5, under ear 5.7, back 9.6, 5.5 and 3.2, thigh 6	Lung
	
	4			Tumor free mouse	None
	
	*5*		dead D219*	Tumor free mouse at day 219	None
	
	6			Tumor free mouse	None
	
	7			cheek 1.4, under eye 3.9, back ears 6.4	None
	
	8			under eye 3.9, under ear 4.7, back 1.5, tail base 2.3	None
	
	9			around eye 6.7	None

The particular aspects of the clinical diagnosis for the control (n = 11) and HB-19 treated (n = 9) RET mice are presented individually for each mouse. Column "Remarks in mice before autopsy" before day 300 gives tumor regression and natural death that occurred in HB-19 treated mouse 2 and 5, respectively. Column "Diagnosis at autopsy" at day 300 gives the location of cutaneous tumors and their size measured with a caliper (the numbers next to each location correspond to the size of the tumor mass in mm), and metastasis occuring at distant locations, i.e., visceral metastasis as well as mediastinal adenopathies or lung metastasis (column "Distant metastasis"). Such non-cutaneous tumors were all chracterized by a large tumor mass (> 4 mm).

* Mouse N°5 died spontaneously at day 219 without symptoms of melanoma.

It is worthwhile to note that two regressions were observed in the HB-19 treated mouse N°2 and concern tumors localized under the eye and thigh (Table 1), while no regression occurred in the control group. Nevertheless, we cannot conclude that these regressions are induced by the treatment although spontaneous regressions are extremely rare in the RET model. Interestingly, at the end of the experiment two out of nine HB-19 treated mice (mouse N°4 and 6) were still tumor free at autopsy, and that mouse N°5 displayed no melanoma symptom at 7.5 months of age, whereas all control mice bore tumors (Table 1). Of note, the absence of cutaneous tumors is extremely infrequent in the RET mouse model. Indeed, in a similar group of 10 month old RET mice in our animal house facility, only one mouse out of 73 was free of cutaneous nodules. Finally, 8 out of 11 control mice had either retroperitoneal metastasis or mediastinal adenopathies, whereas only 3 out of 9 HB-19 treated animals (mouse N°1, 2 and 3) displayed visceral or lung metastasis (Table 1). Therefore, distant metastasis tends to be less frequent in the HB-19 treated compared to the untreated group (Table 1; p = 0.09). We have recently observed that myeloid (CD11b^+^) and T (TCRαβ^+^) cells represent the two major hematopoietic cell populations that infiltrate spontaneous tumors in the RET model (Lengagne et al., in preparation). However, HB-19 treatment did not exert an apparent effect on the proportion of myeloid and T cell populations infiltrating tumors in the RET mice (Table 2).

**Table 2:** HB-19 treatment does not modify the proportion of tumor infiltrating hematopoietic cells

Mice	Mouse Number	% CD45^+ ^cells	% CD11b^+ ^cells	% TCRαβ^+ ^cells
Control	1	3.7	80.4	3.7
	3	1.7	75.8	3.8
	4	2.9	79.8	3.1
	6	2.4	72.2	3.9
	7	1.2	67.1	7.3
	9	2.3	84.3	2.9
	10	1.2	77.0	5.0
	11	2.4	77.0	3.9

HB-19 treated	7	3.8	76.5	5.0
	8	3.3	56.0	6.9

Single cell suspensions were prepared from pools of cutaneous tumors of mice that were still alive at day 300. FACS analysis of hematopoietic cells infiltrating melanoma tumors was carried using pooled suspension of various tumors in control and HB-19 treated mice (see Table 1 and Methods). Tumor infiltrating cells were stained with CD45/CD11b or with CD45/TcRαβ monoclonal antibodies.

During this study, no apparent behavior modifications were noted in response to HB-19 treatment. Moreover, when mice were sacrificed at the end of the study, autopsy revealed no apparent effect on various tissues (including lungs, liver, spleen, and kidney) of HB-19 treated compared to control mice injected with PBS. One out of nine mice treated with HB-19 (mouse N°5) died at day 219 in the course of treatment (Table 1). This mouse displayed no clinical signs of melanoma, thus suggesting a spontaneous death by an unexplained mechanism that might occur although rarely even in control mice.

**Figure 3:**
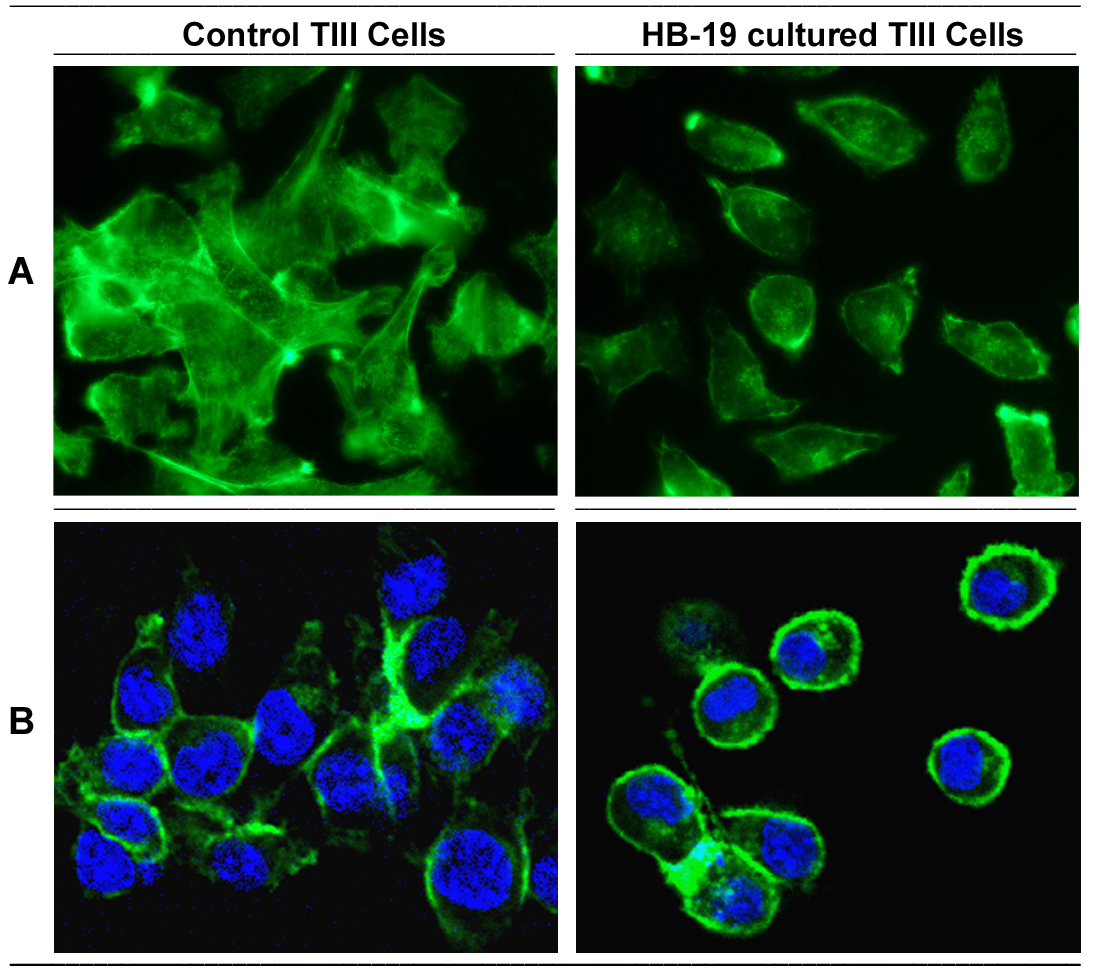
**Restoration of growth contact-inhibition in HB-19 cultured TIII cells**. TIII cells passaged 12-times in the absence or presence of 5 μM of HB-19 were fixed in PFA-Triton, and intracellular actin filaments were stained with FITC-conjugated phalloidin. Immunofluorescence (A) and confocal (B) microscopy was as described in Methods.

**Figure 4:**
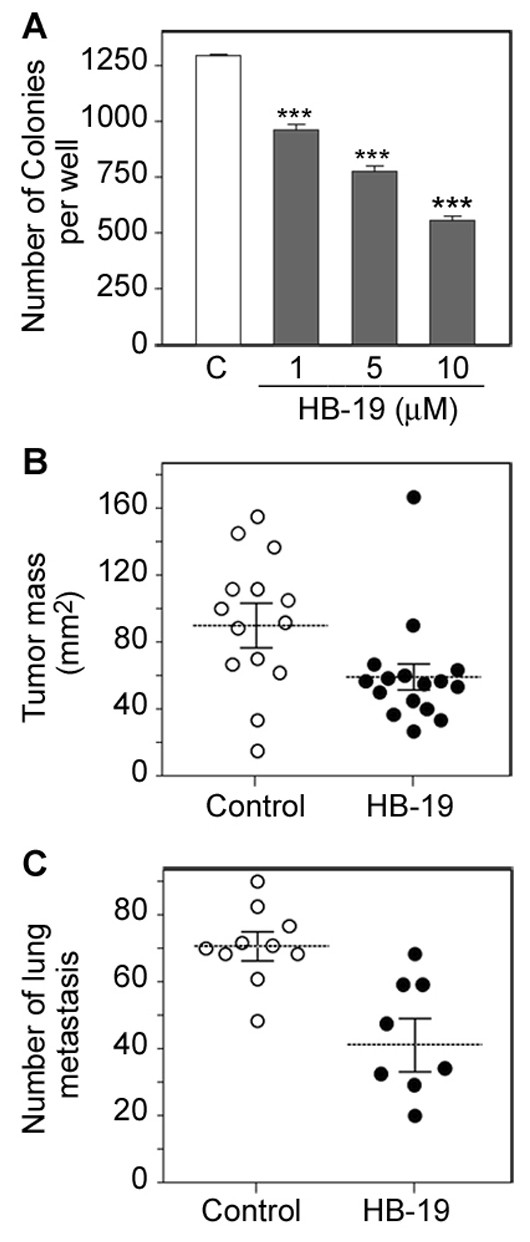
**HB-19 affects the malignant phenotype of melanoma TIII cells**. A. Reduced colony formation in soft agar. TIII cells were cultured for twelve passages in the absence (control, histogram C) or presence of 1, 5 and 10 μM of HB-19 before assay for colony formation in the absence of further HB-19 treatment (***p < 0.001). B. Reduced tumorigenicity in mice. Tumor size was measured fourteen days after transplanting control (open circles, n = 14) or HB-19 precultured (black circles, n = 15) TIII cells. The graph corresponds to the results of two separate experiments. The mice transplanted with HB-19 precultured cells displayed tumors smaller than those treated with control cells (ANOVA, Mann Whitney test, *p *= 0.0276). C. Reduced lung metastasis in mice. Mice injected in the tail vein with TIII cells were treated (closed circles, n = 8) or not (open circles, n = 10) with HB-19, and the number of black lung macro-metastases was determined (ANOVA, Mann Whitney test, *p *= 0.0014).

**HB-19 treatment of melanoma-derived tumor cells impairs their tumorigenic potential**. Multiple passages of TIII cells in the presence of HB-19 resulted in profound effects on cell morphology without affecting significantly the multiplication index of cells. Intracellular staining of actin allowed visualization of such morphological modifications between untreated and HB-19 treated cells (Figure 3). As expected, control TIII cells proliferated without contact inhibition by crawling over each other and with extensions characteristic of migration and/or invasion of tumorigenic cells as compared to the HB-19 treated cells. On the other hand, HB-19 cultured TIII cells appeared to be smaller in size with rounded morphology, thus suggesting that HB-19 treatment could affect their malignant phenotype. In accord with this, the capacity of HB-19 precultured TIII cells to form colonies in soft agar was significantly impaired (Figure 4A). For this purpose, cells were cultured in the absence or presence of different concentrations of HB-19, and then assayed for colony formation without further addition of HB-19. Preculturing cells with HB-19 resulted in a dose dependent reduction of the number of colonies, reaching 56% inhibition when cells were precultured at 10 μM of HB-19.

To assess whether HB-19 can modify the tumorigenic potential of TIII melanoma cells *in vivo*, MT/*ret*^-/- ^mice were injected subcutaneously with control and HB-19 precultured TIII cells. After 14 days, most of the mice that received control TIII cells developed larger tumors than mice that received HB-19 precultured cells (Figure 4B). The mean tumor mass in the control and HB-19 treated group was 91.4 ± 22.5 and 59.1 ± 15.8 mm^2^, respectively. In order to illustrate the action of HB-19 treatment on metastasis, TIII cells were injected intravenously in MT/*ret*^-/- ^mice. The presence of lung metastasis was assessed after 14 days during which time control and HB-19 treated mice received daily intraperitoneal injections of PBS or HB-19, respectively. The results presented in Figure 4C indicate that HB-19 treatment significantly reduced the number of lung metastasis; the mean number of lung macro-metastases in the control and HB-19 treated group was 71 ± 5 and 41 ± 8, respectively.

An altered nuclear nucleolin pattern in cutaneous melanocytic lesions observed by immunohistochemistry has been associated with melanoma progression in patients [33]. Indeed, immunofluorescence laser confocal microscopy studies on the melanoma TIII cells revealed the presence of high number of nucleoli, which could account for the observed nucleolin pattern in melanoma lesions. Using biotinylated HB-19, we demonstrated that HB-19 binds surface nucleolin in a dose dependent manner in the melanoma TIII cells reaching saturation at 4 μM concentration. Following binding, HB-19 is internalized and is concentrated in the cytoplasm without translocation into the nucleus (data not presented). During this process, there is specific reduction of surface/cytoplasmic nucleolin without any apparent effect on the level of nuclear nucleolin or its nucleolar distribution (similar to the data presented in human breast cancer cells [10]). This latter and the lack of an apparent effect on anchorage dependent cell proliferation suggest that the reduced tumorigenicity of HB-19 treated TIII cells is not due to a potential toxic effect of HB-19.

### HB-19 treatment induces down regulation of specific transcripts coding genes associated with tumor cell invasion and metastasis

**Figure 5:**
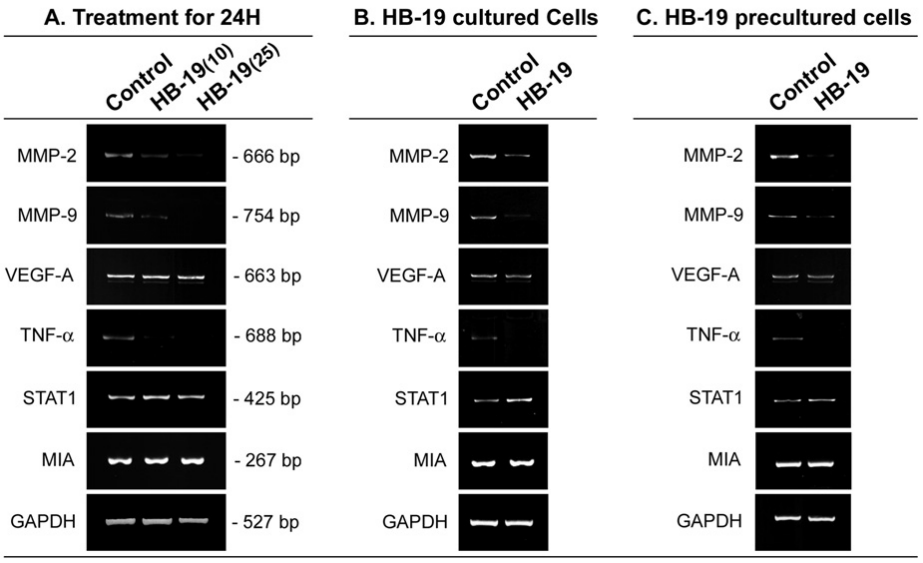
**HB-19 treatment of TIII cells results in the reduction of transcripts coding MMP-2, MMP-9 and TNF-α**. A. Expression of transcripts 24 hours after HB-19 treatment. Two days after seeding, TIII cells were cultured for 24 hours in the absence (Control) or presence of 10 or 25 μM of HB-19. B. Expression of transcripts in control and HB-19 cultured TIII cells (cultured as described in Figure 3). C. Expression of transcripts in control and HB-19 precultured TIII cells (as in section B) that were further passaged 7-times in the absence of HB-19. The expression of specific mRNAs was investigated by RT-PCR using primers for MMP-2, MMP-9, VEGF-A, TNF-α, STAT-1, MIA and GAPDH.

**Figure 6:**
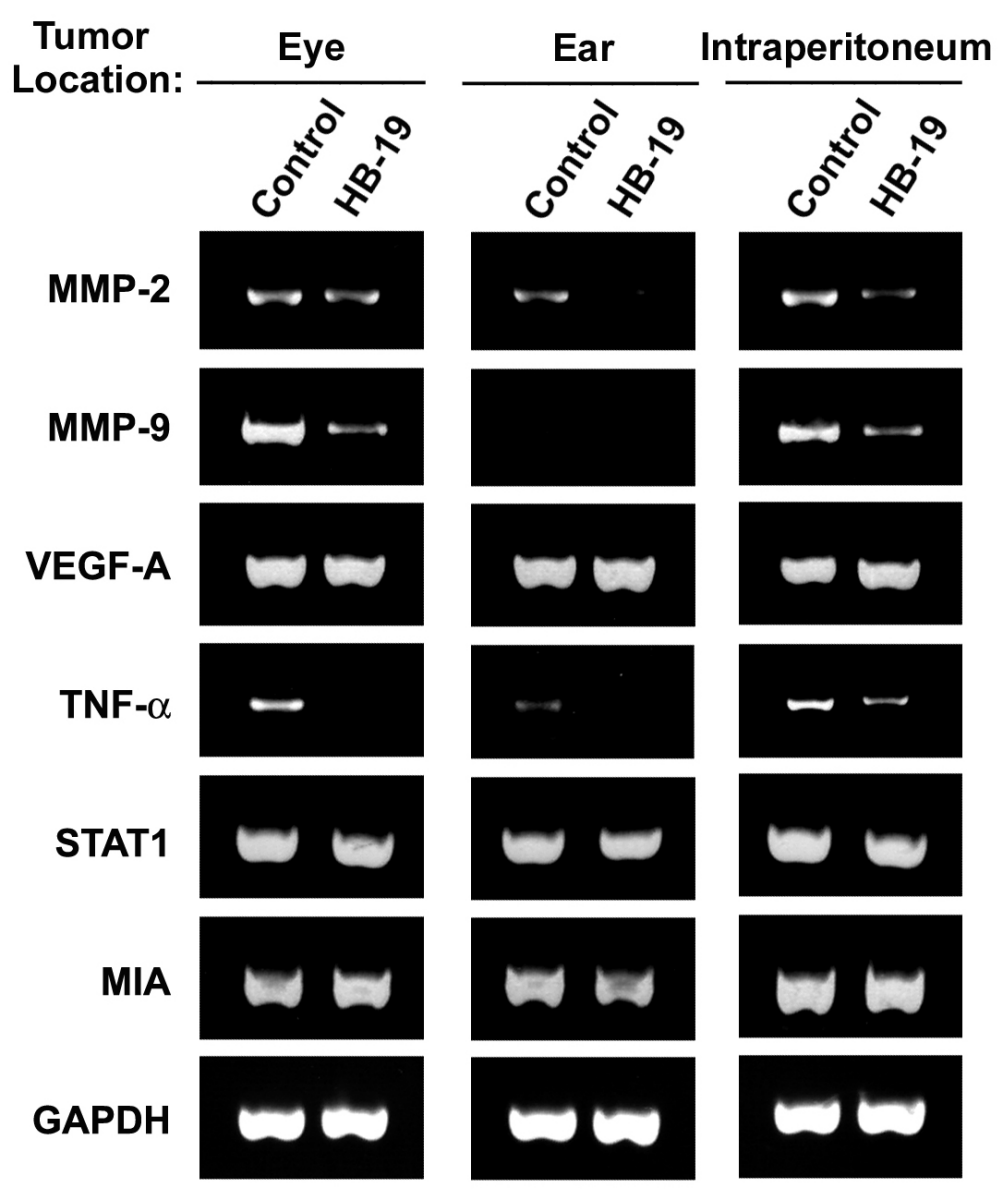
**Reduced expression of transcripts coding MMP-2, MMP-9 and TNF-α in melanoma tumors recovered from HB-19 treated RET mice**. The expression of transcripts coding MMP-2, MMP-9, VEGF-A, TNF-α, STAT-1, MIA and GAPDH was investigated RT-PCR in tumors located at the eye, ear and intraperitoneum of control and HB-19 treated RET mice (as in Figure 5). The eye, ear and intraperitoneal tumors are representative for the firstly diagnosed nodule, a cutaneous nodule and a metastatic nodule, respectively. Equivalent tumor masses at similar locations were considered for comparison.

We investigated the expression of transcripts coding MMP-2 and MMP-9 that degrade components of the extracellular matrix [34], VEGF-A that triggers neovascularization [35], TNF-α that contributes to all stages of the malignant process [36], STAT-1 that regulates major cellular events including tumorigenesis [37], MIA that is associated with progression and metastasis of malignant melanoma [38], and the housekeeping gene GAPDH as a control. Expression of various transcripts was investigated at 3-4 days post seeding of cells, since preliminary experiments indicated that expression of MMP-2 and MMP-9 mRNA is dependent on the cell density as it has been reported previously [39]. Twenty-four hours after HB-19 treatment, the level of transcripts coding MMP-2, MMP-9, and TNF-α was markedly reduced in TIII cells at 10 μM HB-19, whereas these transcripts were completely abolished at 25 μM HB-19. This is a selective effect, since the expression of transcripts coding VEGF-A, STAT1, MIA, and GAPDH seemed not to be affected by HB-19 treatment (Figure 5A). By quantitative RT-PCR, the reduction of transcripts coding MMP-2, MMP-9, and TNF-α was estimated to be > 90 and > 95% at 10 and 25 μM HB-19, respectively (data not shown). We next investigated the level of transcripts in HB-19 cultured TIII cells after twelve passages, and in such HB-19 precultured cells after further seven passages in the absence of HB-19. The expression of transcripts coding MMP-2, MMP-9, and TNF-α was selectively reduced in HB-19 cultured cells, whereas expression of the other genes was not significantly affected (Figure 5B). Interestingly, the expression of transcripts coding MMP-2, MMP-9, and TNF-α remained markedly reduced in HB-19 precultured TIII cells that were further passaged for 7-times in the absence of HB-19 (Figure 5C). Consistent with these *in vitro *results, the expression of transcripts coding MMP-2, MMP-9, and TNF-α is similarly inhibited in the melanoma tumors recovered from HB-19 treated RET mice. A typical example is presented in Figure 6 showing the expression of various genes in tumors recovered from HB-19 treated RET mice at the eye, ear and intraperitoneum compared to tumors recovered from control RET mice at similar locations, respectively.

## Discussion

The results presented herein demonstrate the antitumoral action of HB-19 in the transgenic RET mouse model against development of spontaneous cutaneous melanoma and visceral metastasis. We show that HB-19 treatment of 10 day old mice for a period of 10 months causes a significant delay in the onset and the frequency of large cutaneous tumors compared to untreated control mice (Figure 1). In addition, HB-19 treatment exerts an inhibitory effect on distant metastasis, since the frequency of visceral metastasis was 72% and 33% in the control and HB-19 treated mice, respectively (Table 1). Furthermore, tumor vascularization is reduced significantly in tumors recovered from HB-19 treated compared to untreated control mice (Figure 2). These observations illustrate the dual inhibitory action of HB-19 on tumor cell growth and tumor angiogenesis, consistent with our previous report using athymic nude mice with established human breast tumor cell xenografts [10]. Fogal et al [14] have recently reported that targeting surface nucleolin with an antibody preparation against nucleolin has no significant effect on tumor size or progression while mediating a significant reduction of blood vessel density. This difference in the mechanism of the antitumoral action of anti-nucleolin antibody compared to HB-19 could be due to their mode of interaction with surface nucleolin, since HB-19 binds the C-terminal RGG domain [26] whereas the epitope of the anti-nucleolin antibody is in the N-terminal acidic domain of nucleolin [14].

In the melanoma derived TIII cells, we show that HB-19 treated cells proliferate under contact inhibition and loose partially their tumorigenic potential as demonstrated by impaired colony formation in soft agar, and reduced tumorigenicity and lung metastasis in MT/*ret*^-/- ^mice (Figures 3 and 4). Interestingly, HB-19 treatment induces a specific down regulation of transcripts coding MMP-2, MMP-9, and TNF-α *in vitro *in the TIII cells and *in vivo *in tumors of HB-19 treated RET mice (Figure 5 and 6). Strikingly, the expression of these genes remains down regulated in HB-19 pretreated TIII cells even after seven passages in the absence of HB-19, thus suggesting that HB-19 treatment could trigger differentiation of cultured melanoma cells into a sub-population with somewhat stably reduced malignant phenotype. Consistent with the results observed in melanoma TIII cells, we have shown that HB-19 treatment impairs the tumorigenic potential of several human epithelial tumor cells of different origin, such as breast (MDA-MB-231, MDA-MB-435), prostate (LNCaP), renal (G401), and colon (SW480, SW620) carcinoma (manuscript in preparation). For example, HB-19 treatment of G401 cells leads to restoration of contact inhibition, specific down regulation of several genes associated with tumorigenesis, and marked reduction of tumorigenicity in the nude mice. Therefore, the observed inhibitory action of HB-19 on the melanoma TIII cells is not specific for the rearranged RET-driven melanoma [27,40]. The RET mice express *rfp-ret *hybrid oncogene identified from a recombination event in transfection assays carried on murine NIH 3T3 cells [40]. Consequently, the potential role of RET as a human oncogene remains to be demonstrated. Nevertheless, it is interesting to note that activation of the intrinsic *c-ret *proto-oncogene has recently been correlated with melanoma cell proliferation. Accordingly, the c-RET protein is reported to be expressed in human melanomas while in human malignant melanoma cell lines and in the RET mice there is increased expression of c-RET transcripts [31,41].

We recently reported that HB-19 can trigger rapid and intense membrane Ca^2+ ^fluxes in various types of tumor cells by a mechanism that involves store-operated Ca^2+ ^entry [8]. Consequently, HB-19 could be involved in the activation of signaling pathways leading to regulation of gene transcription. However, the mechanism responsible for selective inhibition of MMP-2, MMP-9 and TNF-α expression in HB-19 treated melanoma cells and tumors remains to be elucidated. Matrix metalloproteinases are extracellular proteinases associated with cancer invasion and metastasis by virtue of degrading components of the extracellular matrix [34], whereas proinflammatory cytokines are indispensable participants in the neoplastic process by orchestrating a tumor-supporting microenvironment [42]. Expression of MMP-2, MMP-9 and TNF-α is strongly linked with malignant tumor progression, angiogenesis and metastasis of various types of cancers [43,44]. Consequently, the selective down regulation of such strategic genes could account, at least in part, for the mechanism of the antitumoral action of HB-19 in RET mice. This is in accord with the results presented in Figure 1 and Table 1 showing a significant delay for the development of spontaneous tumors and the reduced incidence of visceral metastasis in HB-19 treated RET mice compared to the corresponding controls. Although the expression of transcripts coding various isoforms of VEGF-A is not affected in HB-19 treated TIII cells (Figure 5), we have previously shown that HB-19 impairs several VEGF induced endothelial functions involved in angiogenesis by targeting surface nucleolin [10]. By its capacity to block activation of endothelial cells therefore, HB-19 could impair tumor vascularization in RET mice (Figure 2). Taken together, these *in vitro *and *in vivo *studies provide new insights into the mechanism of antitumor action of HB-19, and suggest that several inhibitory pathways could be operating in order to coordinate the delay in the development of melanoma in HB-19 treated RET mice. Recently, it was reported that infiltration of functionally impaired CD8^+ ^T cells, regulatory T cells, tolerogenic dendritic cells and macrophages can occur within metastatic melanoma lesions in patients [45-47]. We found no profound alteration in the proportion of myeloid and T cell populations infiltrating tumors in HB-19 treated and related control RET mice. Consequently, it is unlikely that the inhibitory effect of HB19 treatment is due to the quantitative modulation of the tumor infiltrating immune cells. However, we cannot rule out the potential implication of such hematopoietic cells in the overall antitumoral action of HB-19.

HB-19 treatment significantly delays the development of cancer in RET mice while displaying no toxicity to normal tissue. After binding surface nucleolin, HB-19 enters cells by an active process but it does not cross the nuclear membrane. Consequently, the effect of HB-19 is exerted differentially and specifically via the cell surface expressed nucleolin [10,26]. Although nuclear nucleolin is involved in many aspects of gene expression [1,2], the lack of translocation of HB-19 to the nucleus and nucleolus could account, at least in part, for its lack of toxicity in cultured cells and in animals. By studies on the pharmacokinetic and biodistribution properties of HB-19 in rats, we have demonstrated that after preferential uptake of HB-19 by specific tissues it is eliminated by renal glomerular filtration in the form of HB-19 metabolites [15]. This and the threshold for tissue uptake of HB-19 could prevent prolonged accumulation of HB-19 *in vivo*, which otherwise would lead to toxic effects [15]. Consistent with this, no apparent toxicity was observed in the RET mice that were treated over a period of 10 months with HB-19. The molecular target of HB-19 *in vitro *and *in vivo *is surface nucleolin that is expressed by activated and proliferating cells [2,3,15,48]. In contrast to normal cells however, nucleolin is constantly and abundantly expressed on the surface of tumor cells making them a preferential target for the inhibitory action of HB-19 (Hovanessian et al., submitted).

## Conclusion

The growth and metastasis of solid tumors are dependent on neovascularization in order to provide an appropriate blood supply necessary for tumor cell proliferation and tumor invasion [49]. Although HB-19 treatment failed to prevent the development of spontaneous melanoma in the RET mice, it delayed significantly the onset and frequency of cutaneous tumors, and reduced visceral metastasis and tumor vascularization. Therefore, the inhibitory action of HB-19 on tumor and endothelial cells as well as on metastasis ([10] and the results herein) fulfills the criteria as an efficient and a nontoxic drug for therapeutic intervention in cancer. HB-19 could also be used as an alternative therapy in cancer patients that develop resistance to chemotherapy. Another advantage of HB-19 over traditional anti-cancer drugs is its capacity to bind surface nucleolin in an irreversible manner under physiological conditions [26], making the half-life of tissue associated HB-19 much longer compared to that of any other cancer drug [15]. Finally, its reproducible synthesis, stability in serum and *in vivo *lack of toxicity make HB-19 a unique drug against tumor growth and angiogenesis, thus providing novel therapeutic opportunities in cancer therapy by itself or as an adjuvant therapy in association with current therapeutic interventions on a virulent cancer like melanoma.

## Abbreviations

RET mice: mice expressing constitutively an active form of the *ret *oncogene under the control of the Metallothionein promoter; MMP-2: matrix metalloproteinase-2; MMP-9: matrix metalloproteinase-9; VEGF-A: vascular endothelial growth factor A; TNF-α: tumor necrosis factor alpha; STAT-1: signal transducer and activator of transcription 1; MIA: melanoma inhibitory activity; GAPDH: glyceraldehyde-3-phosphate dehydrogenase

## Competing interests

The authors declare that they have no competing interests.

## Authors' contributions

DEK performed experiments. DD performed experiments. RL performed in vivo treatments and related statistical analysis. BK performed experiments. YHK performed in situ experiments. SN performed in vivo treatments. MG performed in vivo treatments. MK provided the RET mice. JPB synthesized HB-19 and helped to draft the manuscript. JC designed the experiments performed by D.D. and Y.H.K. and helped to draft the manuscript. AGH conceived the overall research plan, designed experiments, and wrote the paper. APB conceived the overall research plan, designed experiments, and wrote the paper. All authors read and approved the final manuscript.

## Pre-publication history

The pre-publication history for this paper can be accessed here:

http://www.biomedcentral.com/1471-2407/10/325/prepub
